# Deep Learning: A Heuristic Three-Stage Mechanism for Grid Searches to Optimize the Future Risk Prediction of Breast Cancer Metastasis Using EHR-Based Clinical Data

**DOI:** 10.3390/cancers17071092

**Published:** 2025-03-25

**Authors:** Xia Jiang, Yijun Zhou, Chuhan Xu, Adam Brufsky, Alan Wells

**Affiliations:** 1 Department of Biomedical Informatics, University of Pittsburgh, Pittsburgh, PA 15206, USA; yijunz317@gmail.com (Y.Z.);; 2 Division of Hematology/Oncology, University of Pittsburgh School of Medicine, Pittsburgh, PA 15213, USA; 3 UPMC Hillman Cancer Center, Pittsburgh, PA 15232, USA; 4 Department of Pathology, University of Pittsburgh and Pittsburgh VA Health System, Pittsburgh, PA 15261, USA

**Keywords:** deep learning, machine learning, grid search, neural networks, breast cancer metastasis, metastatic breast cancer, breast cancer, metastasis, prediction, EHR, clinical

## Abstract

**Background:** A grid search, at the cost of training and testing a large number of models, is an effective way to optimize the prediction performance of deep learning models. A challenging task concerning grid search is time management. Without a good time management scheme, a grid search can easily be set off as a “mission” that will not finish in our lifetime. In this study, we introduce a heuristic three-stage mechanism for managing the running time of low-budget grid searches with deep learning, sweet-spot grid search (SSGS) and randomized grid search (RGS) strategies for improving model prediction performance, in an application of predicting the 5-year, 10-year, and 15-year risk of breast cancer metastasis. **Methods:** We develop deep feedforward neural network (DFNN) models and optimize the prediction performance of these models through grid searches. We conduct eight cycles of grid searches in three stages, focusing on learning a reasonable range of values for each of the adjustable hyperparameters in Stage 1, learning the sweet-spot values of the set of hyperparameters and estimating the unit grid search time in Stage 2, and conducting multiple cycles of timed grid searches to refine model prediction performance with SSGS and RGS in Stage 3. We conduct various SHAP analyses to explain the prediction, including a unique type of SHAP analyses to interpret the contributions of the DFNN-model hyperparameters. **Results:** The grid searches we conducted improved the risk prediction of 5-year, 10-year, and 15-year breast cancer metastasis by 18.6%, 16.3%, and 17.3%, respectively, over the average performance of all corresponding models we trained using the RGS strategy. **Conclusions:** Grid search can greatly improve model prediction. Our result analyses not only demonstrate best model performance but also characterize grid searches from various aspects such as their capabilities of discovering decent models and the unit grid search time. The three-stage mechanism worked effectively. It not only made our low-budget grid searches feasible and manageable but also helped improve the model prediction performance of the DFNN models. Our SHAP analyses not only identified clinical risk factors important for the prediction of future risk of breast cancer metastasis, but also DFNN-model hyperparameters important to the prediction of performance scores.

## Simple Summary

Breast cancer metastasis is a major cause of death for women, and predicting its occurrence is vital for better treatment planning. In this study, the authors developed deep learning models to predict the risk of breast cancer metastasis over five, ten, and fifteen years. One challenge in improving the performance of these models is selecting the best set of adjustable parameters, which can be time-consuming and costly. The study introduces a three-stage grid search method to optimize these parameters while managing limited computational time. The first stage narrows down reasonable ranges for parameters, the second finds the optimal values, and the third refines the models using specific search strategies. The results showed that this method improved the accuracy of predictions by 18.6%, 16.3%, and 17.3% for the different time frames compared to random searches. This approach is valuable for clinicians, as it offers a manageable way to develop better predictive models despite limited resources. Additionally, the study highlights key factors influencing breast cancer metastasis prediction and identifies important parameters for improving model performance. These insights can contribute to more personalized and effective treatment plans for patients.

## 1. Introduction

Electronic health record (EHR) systems have been used in clinical settings for many years. With the help of these systems, researchers, or medical practitioners who work in a clinical environment often have opportunities to access and curate a clinical dataset concerning a group of patients, and sometimes they may consider using such a dataset to build a patient outcome prediction model. For instance, one can use the EHR data collected from a breast cancer patient care center to develop deep learning models that can be used to predict for a patient the risk of future occurrences of breast cancer metastasis.

Breast cancer is a major cancer-related cause of death for women worldwide. Based on the report updated on 13 March 2024 by the World Health Organization (WHO), “Breast cancer was also the most common cancer in women in 157 countries out of 185 in 2022”, and it is also one of the main causes of cancer related death in women worldwide, and “caused 670,000 deaths globally in 2022”. Again, according to the WHO, as of the end of 2020, “there were 7.8 million women alive who were diagnosed with breast cancer in the past 5 years, making it the world’s most prevalent cancer”. Women do not die of breast cancer, rather, they die mainly due to breast cancer metastasis, which can occur years after the initial treatment of breast cancer [1,2]. Predicting a late metastatic occurrence of breast cancer for a patient is important because the prediction can help make more suitable treatment plans for the patient, which may help prevent breast cancer metastasis. Improving our capability of predicting breast cancer metastasis is an important task in breast cancer patient care. Even just a small percentage of improvement can help greatly improve patient quality of life and save lives and care-related costs.

The field of machine learning (ML) and deep learning [3,4,5,6,7,8] has provided us with various AI-based computational methods for conducting predictions. Using these ML methods, we can learn a prediction model automatically from a dataset. However, a prediction model that is developed in such a manner does not always predict well [9]. There are often multiple factors that can affect model performance. For example, the model performance is usually dataset-dependent, that is, the same machine learning method can perform totally differently when it is applied to different datasets [4,9,10]. This phenomenon is perhaps partly because different datasets contain different levels of “signals” that are critical in making correct predictions. When a dataset contains very weak signals, even an advanced method can fail learning a good prediction model. By “signals” we mean the information usable for making predictions, contained in data. A good example of a signal is the so-called correlation between two variables. If two variables are correlated, then one of them can be used to predict the other, and in that case, the former is often called the predictor and the latter is often called the outcome. Sometimes, to curate a dataset that contains sufficient information for learning a good prediction model, we need to collect a lot of data. Generally speaking, the more datapoints (cases) a dataset contains, the more likely it provides sufficient information for learning a good prediction model. That perhaps explains in an aspect why the applications of ML methods are often associated with the term “big data” and a field called data science.

Other than the dataset itself, another important factor for model performance is the value used for an adjustable hyperparameter of a prediction model. All machine learning methods that we used so far have adjustable hyperparameters, but a difference is some methods have more and some have less [4,11,12,13,14]. During the early years of using machine learning methods to carry on real-life prediction tasks, we paid little attention to the selection of a value for an adjustable hyperparameter that is built into a machine learning method [9,10,15]. A normal practice is to use the default value recommended by the developers of the machine learning method or by a machine learning textbook, or at most try a few values that are close to the default value. This may have contributed significantly to the fact that some of the ML methods such as the first generation of the Neural Network reportedly had poor prediction performance [9,10,16].

Deep learning is a machine learning method that has quite some adjustable hyperparameters [17,18,19]. For example, we identified 13 adjustable hyperparameters (see Table 1 and a more detailed description of these hyperparameters in Section 2.3) for the Deep Feedforward Neural Network (DFNN) models that we developed for predicting later occurrences of breast cancer metastasis [4,19]. In a previous study, by conducting machine learning experiments, we found that different value assignments of the adjustable hyperparameters can lead to models with significantly different prediction performance [4]. We used a method called *grid search* [4,20,21,22,23,24] to systematically train and test different DFNN models by changing the values of the set of adjustable hyperparameters [4]. Therefore, in order to do a grid search, we normally preselect a range of ranges for each of the hyperparameters as an input to the grid search.

We now use an example to explain what a grid search does and, in the meantime, introduce a major challenge of conducting a grid search. In this example, we use the number of values given to each of the 13 hyperparameters as shown in the second row of Table 1 below. The number of hidden layers and the number of hidden nodes (neurons) in a hidden layer are the two structural hyperparameters that together determines the structure of a DFNN model. In this example grid search, the number of hidden layers has four different values, that is, the model can contain up to four hidden layers. The number of hidden nodes per hidden layer has 22 different values. Therefore, there could be 22, 484, 10,648, or 234,256 different model structures when the number of hidden layers is configured to be 1, 2, 3, or 4. Thus, we can make a total of 245,410 different models by considering the two structural hyperparameters alone. The total number of possible unique value assignments to the set of the 11 remaining non-structural hyperparameters are the product of the number of values given to each of these hyperparameters, which arrives at 1.7424 × 10^9^. Therefore, considering all 13 hyperparameters, there are in total 4.276 × 10^14^ unique value assignments. What the example grid search does is to train and test 4.276 × 10^14^ DFNN models determined by the 4.276 × 10^14^ different value assignments, one at a time. We call a unique value assignment to the set of hyperparameters of a grid search a *hyperparameter setting*. Note that under each of the hyperparameter settings, there would be k different models trained and tested if the k-fold cross validation (CV) procedure (see the Methods section) is applied. Since we use a 5-fold CV procedure in our grid searches, the number of models trained and tested is five times the number of hyperparameters settings used in these grid searches.

A grid search can be very costly. Based on the grade search experiments we conducted in our previous study [4], the average running time per hyperparameter setting is 117 s for a particular dataset we used. By using this average unit running time, the estimated total running time for the example grid search, as described above, would be 1,586,424,369 years. Apparently, this grid search is not feasible for us unless we use billions of computers to run it parallelly. A grid search is, in general, very time consuming, but computation time can sometimes be resolved by using high-speed computing, which can be bought by money. Therefore, the feasibility issue of a grid search essentially boils down to a financial budget issue. Going back to the scenario that we mentioned at the beginning of this introduction, in which medical researchers or practitioners want to learn a prediction model using their own EHR-based datasets, a normal situation is that the time and funds available are both very limited for conducting a grid search to optimize prediction. We call a grid search for which the money allocation is very limited a low-budget grid search. It is not uncommon to encounter a low-budget grid search in the real-life applications of machine learning methods.

Moreover, the example grid search we described above can only be called a small-scale grid search, in which only a small number of values is given to each of the hyperparameters. But the estimated running time is already hard to manage. Based on this example, we see that the number of values allowed for each of the hyperparameters can only be very small for a low-budget grid search to finish in a foreseeable time. But note that some of the hyperparameters of our DFNN models can take a very large number of different values (in Section 2.3). For example, based on our previous studies [4,19], the range of values we consider for a hyperparameter called *epochs* is from 5 to 2000, which means for each grid search we need to select a very small set of values from the 1996 different values for epochs. As a matter of fact, most of the 13 hyperparameters can take a very large number of values, and some can even take an infinite number of different values within a normal range. It is often a challenging task to select a very small set of values from a large number of values available for each of the hyperparameters like epochs, and in the meantime to ensure the feasibility of a low-budget grid and meet the goal of digging out a better prediction model through the grid search. To our knowledge, there is no standard and good way of doing this. In this study, we have limited time to run grid searches for optimizing our DFNN models that predict the risk of breast cancer metastasis. We therefore introduce what we call a three-stage heuristic grid-search mechanism that we use to manage this challenging task. We describe this mechanism and the experiments we conduct, in which we apply this mechanism, in Section 2, and present and analyze the results of the experiments in Section 3 and Section 4.

## 2. Methods

### 2.1. The DFNN Models

**Figure 1:**
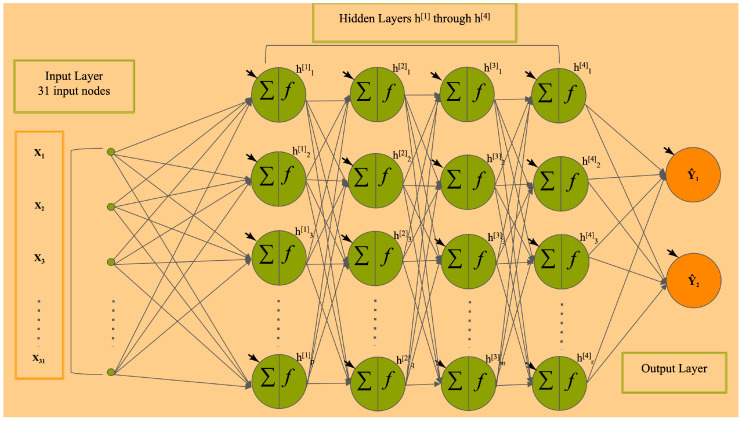
The structure of an example DFNN model we developed [4,19], a fully connected deep feedforward neural network that consists of an input layer, 4 hidden layers, and an output layer.

Deep learning and deep feedforward neural network (DFNN): An Artificial Neural Network (ANN) is a machine learning framework, which is designed to recognize patterns using a model loosely resembling the human brain [25,26]. ANNs can be used for clustering (unsupervised) on unlabeled data or classification (supervised) on labeled data [4]. Deep Neural Networks (DNNs), called deep learning, refers to the use of neural networks composed of more than one hidden layer [3,5,6,7,27]. The DNN has obtained significant success in commercial applications such as voice and pattern recognition, computer vision, and image-based processing [28,29,30,31,32,33,34,35,36,37,38]. However, its power has not been fully explored or demonstrated in applications that are not image-based, such as the prediction of breast cancer metastasis using non-image clinical data. This is due in part to the sheer magnitude of the number of variables involved in these problems, which presents formidable computational and modeling challenges [17,18,39]. We developed the deep feedforward neural network (DFNN) models that predict the risk of a future occurrence of breast cancer metastasis for a patient [4,19] and conduct grid searches to hand these challenges. The DFNN models we developed are fully connected neural networks that do not contain cycles. Figure 1 illustrates, as an example, the structure and the inner connections of the DFNNs that we have developed. The example shown in Figure 1 is a six-layer neural network that contains one input layer, four hidden layers, and one output layer. The 31 input nodes to this neural network represent the 31 clinical features contained in the patient data that we use, and the output layer contains two nodes representing the binary status of 5-year, 10-year, or 15-year breast cancer metastasis. Each node in this model has an activation function (in Section 2.3), represented by *f*(*x*), which decides the node’s individual output value established by the current value of the node. In such a DFNN model, each hidden layer has a certain number of hidden nodes that can be different from the other layers. Both the number of hidden layers and the number of hidden nodes in a hidden layer are two of the set of hyperparameters whose values are subjected to changes during grid searches. These two hyperparameters together with other adjustable hyperparameters for our grid searchers are described in Section 2.3 below.

### 2.2. Datasets

The MBIL method is a Bayesian Network-based method for identifying risk factors (RFs) for an outcome feature, which was applied to three EHR-based clinical datasets concerning breast cancer metastasis, that is, the LSM-5-year, 10-year, and 15-year datasets [40]. These LSM datasets were preprocessed through previous research concerning a clinical decision support system [2] and therefore do not contain missing values. The procedure for preprocessing the EHR-based dataset can be found in this previous study [2]. We use MBIL to retrieve all RFs concerning breast cancer metastasis from the LSM datasets and develop three new datasets according to the RFs: the LSM_RF-5-year, 10-year, and 15-year datasets. Using the LSM_RF-5Year dataset as an example, the 2-step procedure for developing this dataset is as follows: Step 1, applying the MBIL method to the LSM-5Year dataset to retrieve the RFs of 5-year breast cancer metastasis. The original LSM-5-year dataset contains 32 features including a feature called metastasis, which represents the state of having or not having breast cancer metastasis by the 5th year post the initial treatment [40]. In this study, metastasis is the outcome feature, which we also call the target feature, because we are interested in predicting the value of these features using the other features; the remaining 31 features are called predictors. Step 2: remove all predictors that do not belong to the set of RFs found in Step 1 from the LSM-5Year dataset, and all data points of the remaining features form the LSM_RF-5-year dataset. We also follow this 2-step procedure to obtain the LSM_RF-10-year dataset from the original LSM-10-year dataset and the LSM_RF-15-year dataset from the LSM-15-year dataset. All experiments conducted in this study are based on the RF datasets. Table 2 below shows the counts of the cases and predictors included in the three RF datasets. A detailed description of the predictors is included in the Tables S1–S3 of the supplement.

### 2.3. The Three-Stage Grid Search Mechanism and the Experiments

**Figure 2:**
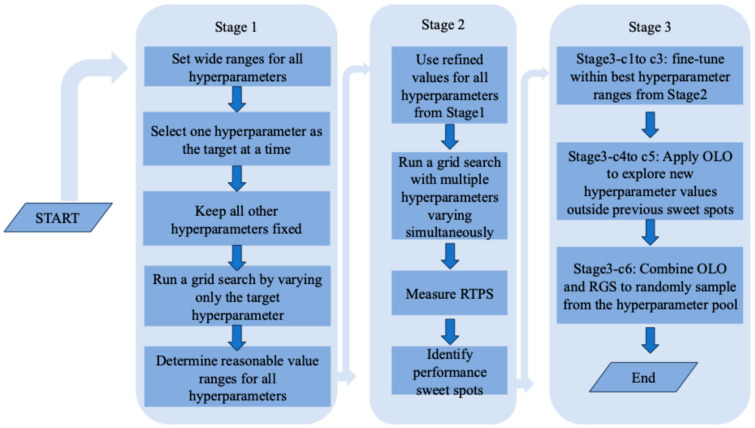
A flowchart for the 3-stage grid search procedure.

In this study, we tune 13 hyperparameters (Table 3) following a heuristic three-stage grid search mechanism for learning the DFNN models. In Stage 1, we focus on learning performance trends and a set of proper values for each of the hyperparameters that can take a very large or an infinite number of different values, such as the epochs and learning rate. The extreme values that can result in performance outliers, that is, the models that perform very poorly, are usually removed from the set of proper values. Stage 2 and Stage 3 grid searches are guided by the set of proper values. In Stage 2, we attempt to estimate the average running time per hyperparameter setting (RTPS) and identify performance sweet spots by conducting a grid search that is guided by the results of the Stage 1 grid searches. The main difference between the Stage 1 and the Stage 2 grid searches is that, in a Stage 1 grid search, we only change the values of a single hyperparameter, and this allows the Stage 1 grid search to train and test models using a large number of different values of the hyperparameter and still finish in an acceptable timeframe. But in a Stage 2 grid search or a Stage 3 grid search, we allow all hyperparameters to take multiple different values. The Stage 2 experiments are important because, based on the results of such a grid search, we can do better in managing the running time of the subsequent Stage 3 grid searches. Specifically, we can estimate the total running time based on the RTPS if we know the input number of hyperparameter settings, and on the other hand, we can computer the input number of hyperparameter settings based the total running time allowed. This ensures that a grid search finishes within an acceptable timeframe and, therefore, is critical to a low-budget and time-sensitive grid search. In Stage 3, we conduct multiple cycles of grid searches to further refining mode prediction performance. A cycle means the entire process of running a grid search from preselecting a set of values for each hyperparameter till finishing all experiments concerning model training and testing scheduled for the grid search. In this study, we conduct 6-cycles of Stage 3 grid searches for each of the three datasets, labeled as Stage3-c1, c2, and up to c6, each, respectively. The hyperparameter settings of the stage3-c1 are determined based on the results of the Stage 2 grid searches, that is, when conducting hyperparameter value selection, we focus on a small number of values that are close to the value used by the best model obtained from the Stage 2 grid searches. We call such a grid search a sweet spot grid search (SSGS), in which the sweet spot is determined by a previous grid search. Stage 3 cycle 1 through 5 are all SSGSs. In Stage 3, we also use our out of the local optimal (OLO) strategy for preselecting the input hyperparameter values. This is because in an SSGS we only use a small range of values that are in the neighborhood of the value used in the best model produced from a previous gird search, then the next best value of a new SSGS will still be within this small range of values, forming a situation that resembles the “local optima” phenomenon. Specifically, in Stage3-c4 and Stage3-c5, we identify a new sweet spot that is outside the “neighborhood” of the previous sweet spot to get out of the potential local optimal formed during the SSGSs. Finally, in Stage3-c6, we apply both OLO and a strategy called the randomized grid search (RGS) that we created. Once the proper set of values for each of the hyperparameters is determined via Stage 1, we will be able to determine all possible unique hyperparameter settings, which we call the pool of hyperparameter settings (PHS). A RGS (Randomized Grid Search) is a grid search that trains and tests models at a set of hyperparameters settings that are randomly picked from a PHS. Specifically, we first randomly selected a set of values for each of the adjustable hyperparameters to produce the input set of hyperparameter settings. This is because the number of possible hyperparameter settings in the pool can be very large and cannot be searched exhaustively with the limited time and low budget that we have. The key point of our RGS strategy is to randomly generate a fixed number of hyperparameter settings following uniform distributions, so that the grid search can be finished within a predictable timeframe and in the meantime every hyperparameter setting in the pool has a chance to be selected. Figure 2 below is a flowchart for demonstrating the three-stage grid search mechanism. Table 3 below contains more detailed information about the 13 hyperparameters of our DFNN models. The specific range of values used in each cycle of our three-stage grid searches are shown in Table S4–S6 of the supplement.

### 2.4. Prediction Performance Metrics and the 5-Fold Cross Validation Process

Our grid searches follow the 5-fold cross validation (CV) mechanism to train and test models at each hyperparameter setting and use an AUC score to measure the prediction performance of a model. AUC stands for the area under the curve of a receiver operator characteristic (ROC) curve that plots the true positive rate against the false positive rate for all possible cutoff values [41]. An AUC score measures the discrimination performance of the model.

To conduct a 5-fold CV, we need to split a dataset prior to grid searches. We use the following procedure to split our datasets: (1) split the entire dataset into a train–test set that contains 80% of the cases and a validation set that contains 20% of the cases. The train–test set will be given to a grid search as the input dataset, and the validation set will be kept aside for a later validation test; (2) divide a train–test set evenly into 5 portions for conducting the 5-fold CV. The division is mostly performed randomly except that each portion should have approximately 20% of the positive cases and 20% of the negative cases to ensure that it is a representative fraction of the dataset. We conduct a 5-fold CV at each hyperparameter setting of a grid search. During a 5-fold CV, 5 different models are generated and tested, each is trained using a unique combination of 4 portions and tested with the remaining portion. Then, 5 AUC scores are produced based on the tests, and the average of these scores is called the *mean_test_AUC*. The mean_test_AUC metric is used by grid searches to measure the model discrimination performance. A top hyperparameter setting selection at the end of a grid search is also based on this metric. A top model is developed by refitting the entire train–test set using the top hyperparameter setting selected by a grid search.

### 2.5. The SHAP Values and Plots

We use the SHAP (Shapley Additive Explanations) values to explain the prediction results of our DFNN models and identify the important features for the predicted future risk of breast cancer metastasis. The SHAP values are established based on the Shapley values, which distribute the payoff by measuring the marginal contribution of individual team members to the outcome of a cooperation game [42,43].

We use the Kernel Explainer provided by the SHAP package [44] to compute the SHAP values for the DFNN models. In order to manage computation time while preserving the integrity of the information contained in data, we process the training dataset using the k-means clustering method. We therefore identify k representative cluster centroids, where k is equal to the number of features in the dataset. These centroids, which epitomize the typical characteristics of the training dataset, are then employed as the background data for the SHAP Kernel Explainer. The background value of a feature is the mean of the corresponding feature values of the k centroids, identified using the k-means clustering.

The formula used to compute SHAP values is as follows:

$$\phi_{i\;}(p)=\sum S\subseteq F\backslash \{i\}\frac{\left|S\right|!\left(\left|F\right|-\left|S\right|-1\right)!}{\left|F\right|!}\;[p(S\cup \{i\})-p(S)]$$

In this formula, *F* denotes the complete set of features, *i* represents the *i*th feature, and *S* represents a subset of *F* not including the *i*th feature, that is, S⊆F\{i}. |*S*| is the size of a subset *S*, and |*F*| is the size of the set *F*. Let *p*(x) represents a model’s prediction outcome, then p(S∪{i}) represents the model’s prediction output when using the features in a subset *S* together with the *i*th feature as the predictors, while p(S) denotes the model’s prediction output when using only the features in *S* as the predictors. The term p(S∪{i})−p(S) therefore reflects the contribution to the model’s prediction output made by the *i*th feature with respect to a subset *S*.

Inspired by LIME [45], the Kernel Explainer generates synthetic samples, which are used as the test cases for a model of interest to compute the SHAP value of a feature. Let us call the feature the *i*th feature. Each of the synthetic samples contains the real values of a subset *S*, taken from the 20% set-aside validation dataset (see Section 2), and the background values for the remaining features in *F*, excluding the *i*th feature. The *i*th feature takes its real value for the synthetic sample that is created for obtaining the p(S∪{i}), and takes its corresponding background value in the synthetic sample that is created to obtain the p(S). Recall that the background values for all features in F are generated using the k-means method as described above.

We also use the SHAP package to generate a SHAP bar plot which ranks the feature importance values of all predictors from high to low. A SHAP feature importance value is the mean absolute SHAP value of all the test cases for the feature of interest. Another type of SHAP plots we show are the so called SHAP summary plots, which not only rank the features by their feature importance values but also show the SHAP value of each individual case that is tested. The SHAP heatmap plots show patterns of groups of instances or features, and the dependence plots explore potential interactive features which jointly affect a SHAP value.

## 3. Results

We followed the three-stage mechanism, described in Section 2, and ran grid search experiments in eight cycles: 1 cycle for Stage 1, 1 cycle for stage 2, and 6 cycles for stage 3, named as Stage3-c1, Stage3-c2, and so on and so forth. For each cycle, we identified groups of top performing models, that is, the top 1, top 5, top 10, top 50, and top 100 models, out of all models that were trained. We then computed the average mean_test_AUC for each group as a measurement of group prediction performance. We also obtained the average mean_test_AUC for all models trained during a cycle of grid searches as the largest group. We compare the group prediction performance side by side of the eight cycles of grid searches for predicting 5-year breast cancer metastasis in Table 4, 10-year in Table 5, and 15-year in Table 6 below.

In addition, to compare the top-performing groups of models among the eight cycles of grid searches, we are also interested in knowing the performance of grid searches in identifying “decent” prediction models. An AUC of 0.5 is often treated as the worst prediction performance score, because it indicates that a model’s prediction capability is equivalent to that of a random guess. What we mean by a “decent” prediction model is a model that scores at least as high as what we call a mid-point score, which is the average of 0.5 and the highest score of all models trained during the corresponding grid searches. So, a “decent” prediction model found in a cycle is a model that has an AUC no less than the mid-point score of the cycle of grid searches. A mid-point group is the group of “decent” prediction models found in a cycle of grid searches. Table 7 below compares the results concerning the mid-point groups of the eight cycles of grid searches.

As previously mentioned, managing the running time of a grid search is a challenging task. Knowing the RTPS, the average time it takes to train models at one hyperparameter setting, can be very useful in various aspects such as pre-estimating the total running time of a grid search, and estimating number of hyperparameter settings that a grid search take in when time we have to run a grid search is limited. During each of the eight cycles of grid searches, we periodically updated the corresponding RTPS based on the results we already obtained at the time and then use it to guide upcoming experiments. Table 8 below shows the final RTPSs in seconds, the total number of hyperparameter settings (TNS), and the total running time (TRT) in hours for each cycle of grid searches we conducted, corresponding to the 5-year, 10-year, and 15-year models each, respectively.

**Figure 3:**
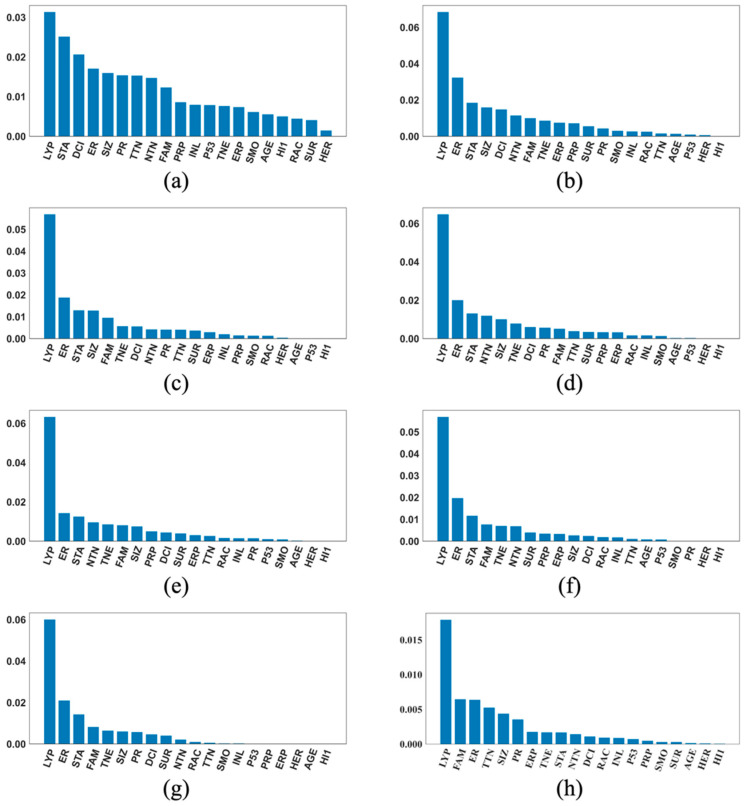
Feature importance plots of the best cycle-wise DFNN-5-year models. (**a**), (**b**), (**c**), (**d**), (**e**), (**f**), (**g**), and (**h**) were generated using the best DFNN models found in Stage 1, Stage 2, Stage 3-c1, …, Stage3-c6, each respectively. **AGE:** age at diagnosis of the disease; **ALC**: alcohol usage; **DCI**: type of ductal carcinoma in situ; **ER**: estrogen receptor expression; **ERP**: percent of cell stain pos for ER receptors; **ETH**: ethnicity; **FAM**: family history of cancer; **GRA**: grade of disease; **HER**: HER2 expression; **HI1**: tumor histology; **HI2**: tumor histology subtypes; **INL**: where invasive tumor is located; **INV**: whether tumor is invasive; **LYP**: number of positive lymph nodes; **LYR**: number of lymph nodes removed; **LYS**: patient had any positive lymph nodes; **MEN**: inferred menopausal status; **MRI**: MRIs within 60 days of surgery; **NTN**: number of nearby cancerous lymph nodes; **PR**: progesterone receptor expression; **PRP**: percent of cell stain pos for PR receptors; **P53**: whether P53 is mutated; **RAC**: race; **REE**: removal of an additional margin of tissue; **SID**: side of tumor; **SIZ**: size of tumor in mm; **SMO**: smoking; **STA**: composite of size and # positive nodes; **SUR**: whether residual tumor; **TNE**: triple negative status in terms of patient being ER, PR, and HER2 negative; **TTN**: prime tumor stage in TNM system.

**Figure 4:**
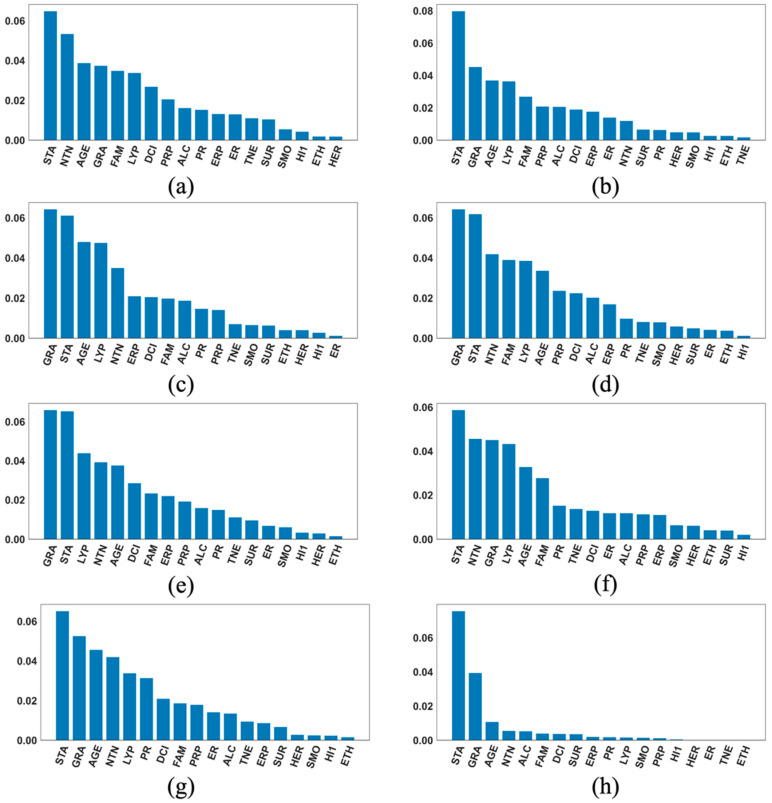
Feature importance plots of the best cycle-wise DFNN-10-year models. (**a**), (**b**), (**c**), (**d**), (**e**), (**f**), (**g**), and (**h**) were generated using the best DFNN models found in Stage 1, Stage 2, Stage 3-c1, …, Stage3-c6, each respectively. **AGE:** age at diagnosis of the disease; **ALC**: alcohol usage; **DCI**: type of ductal carcinoma in situ; **ER**: estrogen receptor expression; **ERP**: percent of cell stain pos for ER receptors; **ETH**: ethnicity; **FAM**: family history of cancer; **GRA**: grade of disease; **HER**: HER2 expression; **HI1**: tumor histology; **HI2**: tumor histology subtypes; **INL**: where invasive tumor is located; **INV**: whether tumor is invasive; **LYP**: number of positive lymph nodes; **LYR**: number of lymph nodes removed; **LYS**: patient had any positive lymph nodes; **MEN**: inferred menopausal status; **MRI**: MRIs within 60 days of surgery; **NTN**: number of nearby cancerous lymph nodes; **PR**: progesterone receptor expression; **PRP**: percent of cell stain pos for PR receptors; **P53**: whether P53 is mutated; **RAC**: race; **REE**: removal of an additional margin of tissue; **SID**: side of tumor; **SIZ**: size of tumor in mm; **SMO**: smoking; **STA**: composite of size and # positive nodes; **SUR**: whether residual tumor; **TNE**: triple negative status in terms of patient being ER, PR, and HER2 negative; **TTN**: prime tumor stage in TNM system.

**Figure 5:**
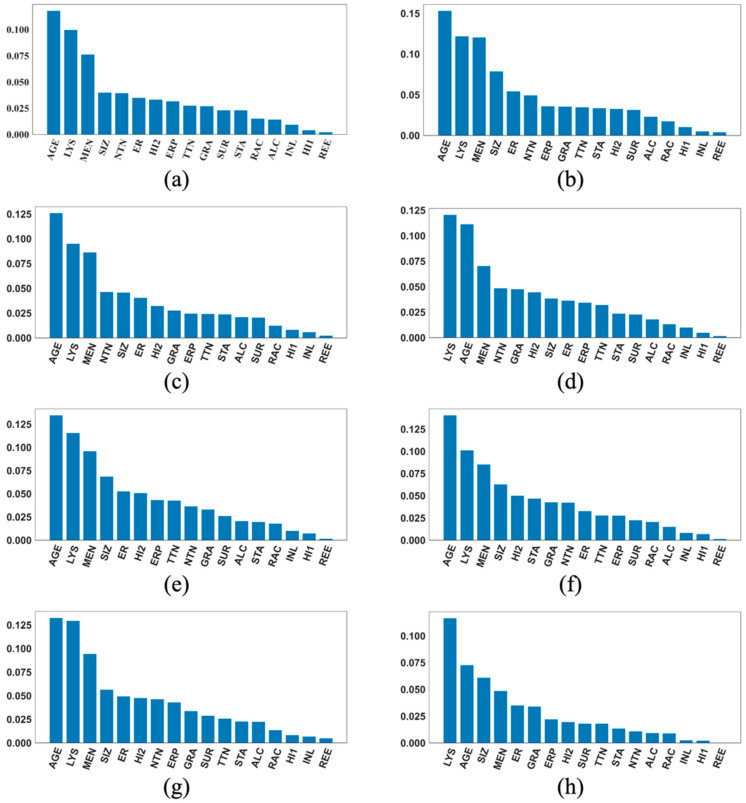
Feature importance plots of the best cycle-wise DFNN-15-year models. (**a**), (**b**), (**c**), (**d**), (**e**), (**f**), (**g**), and (**h**) were generated using the best DFNN models found in Stage 1, Stage 2, Stage 3-c1, …, Stage3-c6, each respectively. **AGE**: age at diagnosis of the disease; **ALC**: alcohol usage; **DCI**: type of ductal carcinoma in situ; **ER**: estrogen receptor expression; **ERP**: percent of cell stain pos for ER receptors; **ETH**: ethnicity; **FAM**: family history of cancer; **GRA**: grade of disease; **HER**: HER2 expression; **HI1**: tumor histology; **HI2**: tumor histology subtypes; **INL**: where invasive tumor is located; **INV**: whether tumor is invasive; **LYP**: number of positive lymph nodes; **LYR**: number of lymph nodes removed; **LYS**: patient had any positive lymph nodes; **MEN**: inferred menopausal status; **MRI**: MRIs within 60 days of surgery; **NTN**: number of nearby cancerous lymph nodes; **PR**: progesterone receptor expression; **PRP**: percent of cell stain pos for PR receptors; **P53**: whether P53 is mutated; **RAC**: race; **REE**: removal of an additional margin of tissue; **SID**: side of tumor; **SIZ**: size of tumor in mm; **SMO**: smoking; **STA**: composite of size and # positive nodes; **SUR**: whether residual tumor; **TNE**: triple negative status in terms of patient being ER, PR, and HER2 negative; **TTN**: prime tumor stage in TNM system.

**Figure 6:**
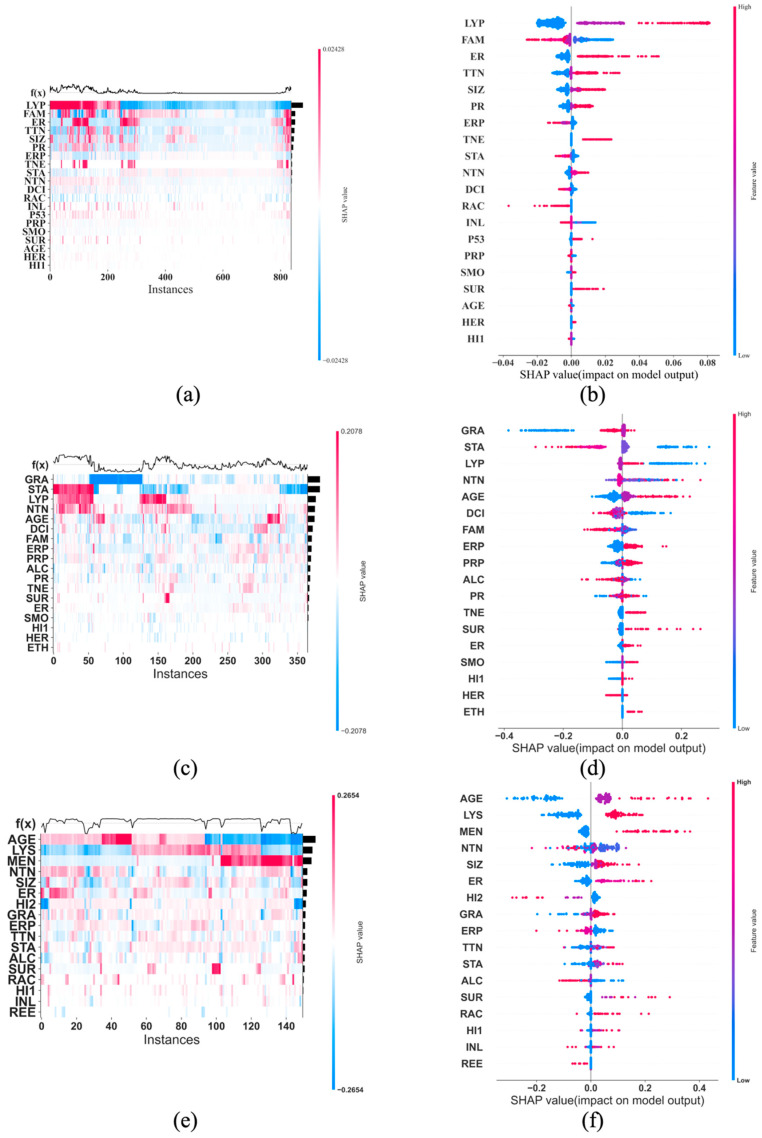
SHAP heatmap and summary plot for the best DFNN-5-year, 10-year, and 15-year model, respectively. (**a**,**c**,**e**) are the SHAP heatmaps produced using the best three DFNN models for predicting 5-year, 10-year, and 15-year breast cancer metastasis, each respectively. (**b**,**d**,**f**) are the SHAP summary plots produced using the same three best models. **AGE**: age at diagnosis of the disease; **ALC**: alcohol usage; **DCI**: type of ductal carcinoma in situ; **ER**: estrogen receptor expression; **ERP**: percent of cell stain pos for ER receptors; **ETH**: ethnicity; **FAM**: family history of cancer; **GRA**: grade of disease; **HER**: HER2 expression; **HI1**: tumor histology; **HI2**: tumor histology subtypes; **INL**: where invasive tumor is located; **INV**: whether tumor is invasive; **LYP**: number of positive lymph nodes; **LYR**: number of lymph nodes removed; **LYS**: patient had any positive lymph nodes; **MEN**: inferred menopausal status; **MRI**: MRIs within 60 days of surgery; **NTN**: number of nearby cancerous lymph nodes; **PR**: progesterone receptor expression; **PRP**: percent of cell stain pos for PR receptors; **P53**: whether P53 is mutated; **RAC**: race; **REE**: removal of an additional margin of tissue; **SID**: side of tumor; **SIZ**: size of tumor in mm; **SMO**: smoking; **STA**: composite of size and # positive nodes; **SUR**: whether residual tumor; **TNE**: triple negative status in terms of patient being ER, PR, and HER2 negative; **TTN**: prime tumor stage in TNM system.

Figure 3, Figure 4 and Figure 5 each contains eight SHAP feature importance plots generated using the eight best DFNN models, each obtained from one of the eight cycles of grid searches, for predicting 5-year, 10-year, and 15-year breast cancer metastasis, respectively. In such a plot, each feature’s mean absolute SHAP value is shown on the *Y*-axis, and the feature names are shown on the *X*-axis. Figure 6 contains two types of SHAP plots, the SHAP heatmaps (Figure 6a,c,e) and SHAP summary plots (Figure 6b,d,f), produced using the three best DFNN models for predicting 5-year, 10-year, and 15-year breast cancer metastasis, each, respectively. Each of the three best Models is selected out of all models trained in the eight cycles of grid searches. A SHAP heatmap details the SHAP value distribution for all features, allowing the revelations of value patterns of grouped cases and features. In such a plot, features are ordered by their SHAP feature importance values from high to low as shown by a black bar plot to the right of the heatmap, and cases are grouped by the similarity of their SHAP impact based on hierarchical clustering. The notation *f*(*x*) represents the distribution of the model’s predictions. The heatmap plots as shown in Figure 6 were produced using the default hierarchical clustering method included in the SHAP package [44]. In companion with a SHAP heatmap, a SHAP summary plot not only shows the ordered average impact of all features but also shows the specific SHAP values of the test cases with respect to each value of a feature. Therefore, such a plot reflects how a value of a feature influences the prediction. The summary plots in Figure 6 were also generated using the SHAP package.

**Figure 7:**
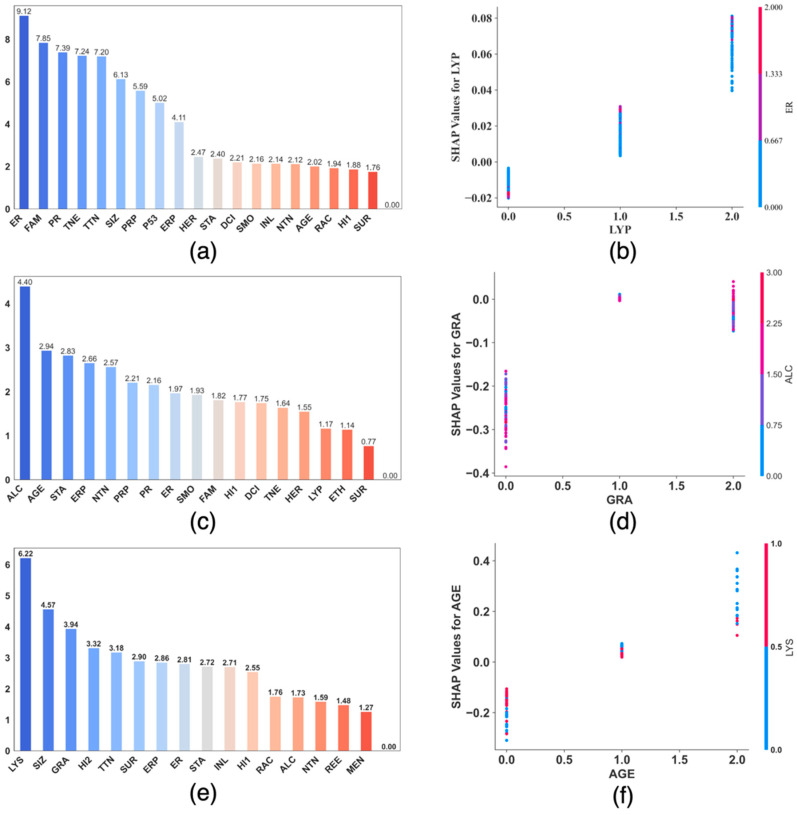
Correlation bar plot and independence plot with interaction visualization for the best DFNN-5Year (**a**,**b**), DFNN-10-year (**c**,**d**), and DFNN-15-year (**e**,**f**) model, each, respectively. **AGE**: age at diagnosis of the disease; **ALC**: alcohol usage; **DCI**: type of ductal carcinoma in situ; **ER**: estrogen receptor expression; **ERP**: percent of cell stain pos for ER receptors; **ETH**: ethnicity; **FAM**: family history of cancer; **GRA**: grade of disease; **HER**: HER2 expression; **HI1**: tumor histology; **HI2**: tumor histology subtypes; **INL**: where invasive tumor is located; **INV**: whether tumor is invasive; **LYP**: number of positive lymph nodes; **LYR**: number of lymph nodes removed; **LYS**: patient had any positive lymph nodes; **MEN**: inferred menopausal status; **MRI**: MRIs within 60 days of surgery; **NTN**: number of nearby cancerous lymph nodes; **PR**: progesterone receptor expression; **PRP**: percent of cell stain pos for PR receptors; **P53**: whether P53 is mutated; **RAC**: race; **REE**: removal of an additional margin of tissue; **SID**: side of tumor; **SIZ**: size of tumor in mm; **SMO**: smoking; **STA**: composite of size and # positive nodes; **SUR**: whether residual tumor; **TNE**: triple negative status in terms of patient being ER, PR, and HER2 negative; **TTN**: prime tumor stage in TNM system.

We further analyze and demonstrate the interpretation of a model’s prediction using the SHAP dependence plots with interaction visualization, as shown in Figure 7. To generate each of the subfigures in Figure 7, we used the best model found from all models trained during the corresponding eight cycles of grid searches. Figure 7a,c,e are the correlation bar plots for the 5-year, 10-year, and 15-year predictions each, respectively, which rank the association strength between each feature and the SHAP values of the most important feature identified using the SHAP feature importance. Using Figure 7e, the figure for the 15-year prediction as an example, since the most import feature based on the SHAP feature importance value (see Figure 6e or Figure 6f) is AGE (*age_at_diagonosis*), Figure 7e shows that the feature that is mostly correlated with the SHAP impact of AGE is LYS (*lymph_node_status*). Figure 7b,d,f are the corresponding dependence plots with interaction visualization, which demonstrate the interaction between the most import feature and the feature that is mostly correlated with the impact of the most important feature. Again, using the 15-year as an example, since LYS is mostly correlated with the SHAP impact of the most important feature AGE, as shown in Figure 7e, Figure 7f visualizes the joint effect of AGE and LYS. In Figure 7f, with respect to each individual case, represented as a colored dot, the value of the most important feature, AGE, is plotted on the *X* axis, the SHAP value for the most important feature is shown on the *Y*-axis, and the color shows the value of LYS, the feature that is mostly correlated with the SHAP impact of AGE.

In the stage3-c6 grid searches, we used the RGS strategy (see Section 2), which allows us to use a broad range of values for each of the hyperparameters (see Tables S4–S6); therefore a large number of different hyperparameter settings were used in these grid searches. Specifically, in this cycle, 70,568, 105,500, and 93,000 different hyperparameter settings were used to train models for predicting 5-year, 10-year, and 15-year breast cancer metastasis, each, respectively. Since the information about each hyperparameter setting and the corresponding mean_test_AUC from the 5-fold CV process are both recorded in our grid-search output, we were able to conduct SHAP analyses using these output as new data to identify the important hyperparameters to the predicted mean_test_AUC scores. In these SHAP analyses, each of the DFNN model hyperparameters was treated as a predictor (a feature), and the mean_test_AUC was treated as the outcome variable.

Our procedure for conducting this unique application of the SHAP analyses is as follows: (1) process the grid search results so that they can be used as the input dataset of the SHAP analyses. For example, we recoded free-text-like values of a categorical hyperparameter such as *kinit* (*kernel initializer*) with digits. Using kinit as an example, we converted the value *constant* to 0, *glorot_normal* to 1, *glorot_uniform* to 2, *he_normal* to 3, and *he_uniform* to 4; (2) use the k-means algorithm to cluster the input dataset into n clusters, n is corresponding to the number of hyperparameters. Then, use the centroid of each cluster to serve as the background data required by the SHAP Kernel Explainer (see the Methods); (3) train a prediction model using 80% of the input data. For our analysis specifically, we trained a prediction model using the Random Forest method with 100 estimators; (4) calculate the SHAP values using the 20% saved data and the prediction model obtained in (3), and then compute SHAP importance values for the features (the hyperparameters).

**Figure 8:**
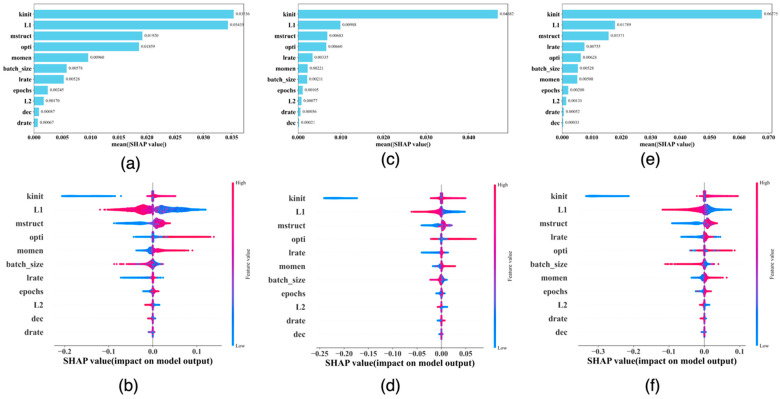
The SHAP importance and summary plots concerning important hyperparameters for explaining predicted mean_test_AUCs based on results of DFNN-5-year (**a**,**b**), 10-year (**c**,**d**), and 15-year (**e**,**f**) models, respectively. **AGE**: age at diagnosis of the disease; **ALC**: alcohol usage; **DCI**: type of ductal carcinoma in situ; **ER**: estrogen receptor expression; **ERP**: percent of cell stain pos for ER receptors; **ETH**: ethnicity; **FAM**: family history of cancer; **GRA**: grade of disease; **HER**: HER2 expression; **HI1**: tumor histology; **HI2**: tumor histology subtypes; **INL**: where invasive tumor is located; **INV**: whether tumor is invasive; **LYP**: number of positive lymph nodes; **LYR**: number of lymph nodes removed; **LYS**: patient had any positive lymph nodes; **MEN**: inferred menopausal status; **MRI**: MRIs within 60 days of surgery; **NTN**: number of nearby cancerous lymph nodes; **PR**: progesterone receptor expression; **PRP**: percent of cell stain pos for PR receptors; **P53**: whether P53 is mutated; **RAC**: race; **REE**: removal of an additional margin of tissue; **SID**: side of tumor; **SIZ**: size of tumor in mm; **SMO**: smoking; **STA**: composite of size and # positive nodes; **SUR**: whether residual tumor; **TNE**: triple negative status in terms of patient being ER, PR, and HER2 negative; **TTN**: prime tumor stage in TNM system.

The results of the SHAP analyses concerning DFNN model hyperparameters are shown in Figure 8 below. Figure 8a,c,e are the SHAP feature importance plots concerning the hyperparameters, generated using the DFNN model grid-search output for predicting 5-year, 10-year, and 15-year breast cancer metastasis, each, respectively, and Figure 8b,d,f are the corresponding SHAP summary plots.

## 4. Discussion

Based on Table 4, for predicting 5-year breast cancer metastasis, the best performing model comes from Stage3-c6, with a mean_test_AUC that is higher than the average mean_test_AUC of all models trained in this cycle by 18.6%. Since we applied the RGS strategy in the Stage3-c6 grid searches, the average mean_test_AUC of all models trained in this cycle reflects the expected model performance when a hyperparameter value is randomly selected from a set of proper values. Table 5 shows that for predicting 10-year breast cancer metastasis, the best performing model comes also from stage 3 but is in cycle 3, with a mean_test_AUC that is higher than the average mean_test_AUC of all models trained in Stage3-c6 by 16.3%. For predicting 15-year breast cancer metastasis, the best performing model is found in stage3-c1, with a mean_test_AUC that is higher than the average mean_test_AUC of all models trained in Stage3-c6 by 17.3%.

Table 4, Table 5 and Table 6 also show that the three-stage grid search mechanism overall works as expected due to the following reasons: Firstly, all best models are found in the Stage 3 grid searches. This is consistent with our goal of further refining grid searches in Stage 3 by building upon the preparation work from the Stage 1 and Stage 2 grid searches. Secondly, we conducted SSGSs in Stage3-c1 through Stage3-c3 grid searches, focusing on refining prediction perform based on the “sweet spot” derived from the results of its previous cycle. As expected, both Table 4 and Table 5 show a steady increase in terms of both the best and group-average model performance across these three cycles of grid searches. Thirdly, we used the RGS strategy in Stage3-c6, in which we allowed a hyperparameter to have a chance of taking any value in its proper set of values, determined based on Stage 1 grid searches. We reckon that such a grid search may accidently identify a best model because it has more “freedom” in choosing its hyperparameter value combinations, but its group model performance may not be as good because the pure randomness in selecting hyperparameter values can also result in very bad models. This is indeed reflected in our results as shown in Table 4 through Table 6.

In Table 4, Table 5 and Table 6, we not only compare the prediction performance of the best models but also compare group model performance among the five groups including top 5, 10, 50, 100, and “All”. The group performance comparisons reflect somewhat the characteristics of the grid searches of the different stages, in which different strategies were applied. As seen in Table 4 through Table 6, Stage 1 grid searches tend to have a low “All” model performance. This is perhaps because in Stage 1 we ran grid searches that focused on one hyperparameter at a time and, therefore, we were able to test a broad range of hyperparameter values. The extreme values used for a hyperparameter could lead to very bad models, which help drag down the average score of all models. Stage 2 overall does better than Stage 1 in group model performance, this is perhaps because the hyperparameter values used in stage 2 were selected from the set of proper values resulting from Stage 1. Table 4, Table 5 and Table 6 also show that Stage 3 Cycle 1 through Cycle 5 tend to do better than both Stage 1 and Stage 2, and the explanation mostly lies in the fact that we conducted SSGSs in these cycles, in which the hyperparameter values used are “in the neighborhood” of some best values found in previous grid searches. The results seem to suggest that, in terms of model performance, if one model does well, then its neighbors tend to do well also. In addition, Cycle 4 and 5 tend to do worse than Cycle 1 through 3 perhaps due to the OLO strategy. Our results also show that Stage 3 Cycle 6 grid searches are competitive in terms of group model performance except for the “All” model group. This indicates that the RGS strategy is competitive in terms of identifying top performing models, but the average performance of all models is brought down by performance outliers resulted from the pure randomness of hyperparameter value selection.

We trained hundreds of thousands of models at each of the eight cycles (see Table 8, and recall that 5 models were trained at each hyperparameter setting due to the 5-fold CV). Out of such a large number of models, how many of them are decent prediction models? An answer to this question may help further characterize the performance of grid searches. Table 7 shows the summary data that we derived from the output of our grid searches to answer this question. We described what we meant by a decent model in the Result section. Based on the CHS/TNS ratios in Table 7, Stage 3 Cycle 1 through Cycle 5 grid searches, that is, the so called SSGSs, did the best in terms of searching for a decent prediction model. The models trained by each of these five cycles are 100% or close to 100% decent, and this is true regardless which dataset was used. We also notice that the Stage 3 Cycle 6 grid searches produced the worst results in terms of identifying decent models, perhaps also due to the randomness of the hyperparameter value selection, required by the RGS strategy.

As described in the Introduction, time management has always been a critical issue in grid searches, especially in low-budget ones. Based on Table 8, Stage 1 and the SSGSs in Stage 3, especially the Cycle 1 through Cycle 4, tend to link to a relatively low RTPS, while the Stage 3 Cycle 6 for which we used the RGS strategy and Stage 2 tend to give a relatively high RTPS. By consulting Table S4 through Figure S6, we found that the values of a hyperparameter called the number of hidden nodes in a hidden layer seem to positively correlate with the RTPS, because the value changes in this hyperparameter can well explain the changes in RTPS. For example, the highest RTPS that we see comes from Stage 3 Cycle 6, which can be explained by the range of high values (550–800) of this hyperparameter used in this cycle. In another example, we notice that Stage 1 allows this hyperparameter to take the highest value 1005 but only has the second highest RTPS. This can be explained by the broadest range of values (1 to 1005) that this hyperparameter takes in Stage 1, which should give an average value that is lower than the values used in Stage 3 Cycle 6. From Table 8, we also notice that within the same cycle of grid searches, where the same search strategy was used, the RTPSs for the three different datasets are also significantly different. The RTPS is the highest for the 5-year dataset, and lowest for the 15-year dataset. Based on Table 2, a significant difference among the three datasets is the size of a dataset, that is, the number of cases (also called data points in machine learning) contained in a dataset. The 5-year dataset contains the largest number of cases, while the 15-year dataset contains the lowest number. Table 2 and Table 8 together reveal a positive correlation between the number of data points contained in a dataset and the RTPS of a grid search.

Based on Figure 3, Figure 4 and Figure 5, the important features in the best models are quite consistent among the eight cycles. A variable called LYP (*lymph_nodes_positive*) is found to be the most important feature for predicting 5-year breast cancer metastasis in all eight best models shown in Figure 3. In addition, Figure 3 also demonstrates that LYP is far more important than the other features, and this is consistently demonstrated by the SHAP importance plots of almost all the best models. According to our knowledge about cancer, it seems to make sense that the number of positive lymph nodes is a good indicator for breast cancer metastasis at diagnosis and, therefore, a good indicator for the risk of cancer recurrence in the near future. Based on Figure 3, the top three important features for predicting 5-year breast cancer metastasis also include ER (*estrogen_receptor_expression*) and STA (*stage*), with ER to be the second most important feature voted by five out of the eight best models, and STA to be the third most important feature voted by six out of the eight best models.

According to Figure 4, the top three most important features for predicting the risk of 10-year breast cancer include STA, identified as the most important in five out of the eight best models, GRA (*grade*), identified as the most important by three out of the eight best models, and AGE (*age_at_diagnosis*), identified as the number three important by five out of the eight best models. Unlike the situation where one feature mostly dominates as seen in the 5-year DFNN models, we see that more than one feature dominate in most of the importance plots. Based on Figure 5, the top three important features for predicting the risk of 15-year breast cancer metastasis are AGE, LYS (*lymph_nodes_status*) and MEN (*menopausal_status*). We also notice that in seven out of the eight best models, these three top features together dominate the overall predicted risk of breast cancer metastasis.

Lymph node status, estrogen positivity, and age are known clinical predictors for 5-year BCSS (breast cancer specific survival) [46,47]. Stage, grade, and age are also known clinical predictors for 10-year BCSS [46,48]. Finally, age, lymph node stage, and menopausal status are predictors of 15-year BCSS [46,48].

Although the feature importance figures (Figure 3, Figure 4 and Figure 5) identify the top feature(s) that dominate(s) the overall importance of the predicted results, they do not show the relationships among the features in terms of their impact. The SHAP heatmaps as shown in Figure 6 perhaps do better in this regard. For example, Figure 6e, a SHAP heatmap plot, reveals color patterns in terms of both features and cases, which indicate similar influences of the grouped members on the model’s prediction. However, the heatmap plots do not show whether the features interact to have a joint effect on prediction.

An interactive effect is an additional effect after subtracting the individual effect from each of the interactive features. A SHAP dependence plot with interaction visualization such as the ones shown in Figure 7 carries more information in terms of a joint contribution between two features. For example, Figure 7e shows that LYS is strongly correlated with the impact from AGE, which is the most important feature for predicting the risk of 15-year breast cancer metastasis (see Figure 5f and Figure 6e). Figure 7f demonstrates that even at the same value of AGE, the SHAP values distribute differently at different values of LYS, confirming that AGE and LYS, the top two most influential features for predicting the risk of 15-year breast cancer metastasis, indeed interact to contribute to the prediction. More specifically, based on Figure 7f, when the value AGE is 0 (for patients who are 0 to 49 years old), it has overall a negative impact on the predicted risk, when the value of AGE is 1 (for patients who are 50 to 69 years old), it has an overall positive but low impact on the predicted risk, when AGE is 2 (for patients who are above 70 years old), it has a significantly increased positive impact on the predicted risk. In addition, Figure 7f reveals that at low ages, LYS tends to be positively correlated with the SHAP impact of AGE, while as age increases, LYS tends to be negatively correlated with the SHAP impact of AGE. In terms of AGE and LYS, LYS is known to clinically affect DFMS (distant metastasis free survival) as above. Age may be important as older women either (1) die of causes other than breast cancer over a 15-year period, and (2) younger women have a longer lifespan to develop distant disease [49]. Additionally older women tend to be undertreated with adjuvant therapy as opposed to younger women [50].

As shown by Figure 8a,c,e, the hyperparameter that has the highest overall impact on the predicted mean_test_AUC is kinit, which is used to assign the initial weights of some regression functions used in gradient descent, a key process in deep learning. This is a bit out of our expectation, because we originally thought for a deep learning, the depth of the network, which is reflected in a hyperparameter called mstruct should be the most important feature. The importance of kinit perhaps comes from its influence on gradient descent. An inappropriate value assignment of kinit can cause divergent learning, which should have a negative impact on model performance. The summary plots as shown in Figure 8b,d,f, reveal that the value of kinit is positively correlated with its SHAP value, which seems to suggest that he_uniform, the highest value of kinit, has the highest positive impact on the predicted mean_test_AUC of a DFNN model. This may help explain that he_uniform was often associated with a good DFNN model, as seen in some preliminary experiments that we conducted [4].

Figure 8 also demonstrates that the second most important feature in terms of the predicted mean_test_AUC is L1, a coefficient of the LASSO regularization, which can help select features and therefore control model sparsity during training [51]. The summary plots in Figure 8 suggest a negative correlation between the values of L1 and its SHAP values. Mstruct ranks number three in terms of its importance to the predicted mean_test_AUCs. It is a two-dimensional hyperparameter for our DFNN models, consisting of two hyperparameters: the number of hidden layers and the number of hidden nodes in a hidden layer. The summary plots in Figure 8 reveal a positive correlation between mstruct and its SHAP values, indicating that the complexity of a neural network structure may have a positive impact on the predicted mean_test_AUCs. This may help explain the observed success of deep neural networks in contrast to the “shallow” neural networks, namely, the first-generation one-hidden layer neural networks. Finally, we also notice from Figure 8 that the rankings of the top three most important features are quite consistent in all plots. This may indicate that the most important adjustable hyperparameters for the DFNN models are not so dependent on a dataset. However, since this is the first time, as far as we know, that a SHAP analysis is used to explain the role of a hyperparameter as a feature in a predicted mean_test_AUC, the implications of the kind of results shown in Figure 8 are subjected to further explorations.

## 5. Conclusions

Our grid searches improve the risk prediction of 5-year, 10-year, and 15-year breast cancer metastasis by 18.6%, 16.3%, and 17.3%, relatively to the average mean_test_AUC of all corresponding models, for which the value of each hyperparameter was randomly picked from a set of reasonable values. Our result analyses characterize grid searches in terms of their capabilities of identifying groups of top performers and “decent” models. The heuristic three-stage mechanism worked effectively. It enables us to finish the low-budget grid searches within the expected timeframe. The results of the grid searches are consistent with what we expected, that is, all the best performing models are found in Stage 3, the final model-refining stage. The local cycles of SSGSs in Stage 3 overall show steadily increased performance not only in terms of the best models, but also in different groups of models. The SSGS Strategy significantly beats the RGS strategy in terms of the average performance of all models and percentage of “decent” models found in all models, but the RGS is quite competitive with the SSGS in identifying best models. The unit grid search time is positively correlated with both the number of hidden nodes in a hidden layer and the number of data points contained in a dataset. The results also suggest that a model with a set of hyperparameter values “close to” the set of values of a good prediction model tends to be a good model. The SHAP analyses not only reveal the features with high impact on the predicted risk of breast cancer metastasis but also help identify pairs of interactive features with regard to SHAP values. Finally, a unique SHAP application demonstrates that the kernel initializer, L1, and mstruct are the top three most important hyperparameters to the predicted mean_test_AUCs.

## Figures and Tables

**Table 1:** The number of values for each of the adjustable hyperparameters of a DFNN model, used in the example grid search.

Hyperparameter Name Acronym *	NHL	NHN	AF	FI	O	LR	M	ID	DR	E	BS	L1	L2
Number of Values	4	22	4	5	5	10	4	11	3	12	11	10	10

* NHL: number of hidden layers; NHN: number of hidden nodes; AF: activation function; KI: kernel initializer; O: optimizer; LR: learning rate; M: momentum; ID: iteration-based decay; DR: dropout rate; E: epochs; BS: batch_size.

**Table 2:** Case and predictor counts of the three LSM_RF datasets.

	Total # of Cases	# Positive Cases	# Negative Cases	# of Predictors
LSM-RF-5year	4189	437	3752	20
LSM-RF-10year	1827	572	1255	18
LSM-RF-15year	751	608	143	17

**Table 3:** The set of adjustable hyperparameters and their ranges of values considered for our grid searches.

Hyperparameter	Description	Values
Number of hidden layers	The depth of a DNN	1, 2, 3, 4
Number of hidden nodes in a hidden layer	Number of neurons in a hidden layer	1–1005
Activation function	It determines the value to be passed to the next node based on the value of the current node	Relu, Sigmoid, Softmax, Tanh
Kernel initializer	Assigning initial values to the internal model parameters	Constant, Glorot_normal, Glorot_uniform, He_normal, He_uniform
Optimizer	Optimizes internal model parameters towards minimizing the loss	SGD, Adam, Adagrad, Nadam, Adamax
Learning rate	Used by both SGD and Adagrad	0.001~0.3
Momentum	Smooths out the curve of gradients by moving average. Used by SGD.	0~0.9
Iteration-based decay	Iteration-based decay; updating learning rate by a decreasing factor in each epoch	0~0.1
Dropout rate	Manage overfitting and training time by randomly selecting nodes to ignore	0~0.5
Epochs	Number of times model is trained by each of the training set samples exactly one	5~2000
Batch_size	Unit number of samples fed to the optimizer before updating weights	1 to the # of datapoints in a dataset
L1	Sparsity regularization;	0~0.03
L2	Weight decay regularization: it penalizes large weights to adjust the weight updating step	0~0.2

**Table 4:** Comparison of group prediction performance in average mean_test_AUCs for the DFNN-5-year models.

Group	Results	Stage1	Stage2	Stage3-c1	Stage3-c2	Stage3-c3	Stage3-c4	Stage3-c5	Stage3-c6
Top1	best	0.75301	0.74812	0.75024	0.75066	0.75418	0.75386	0.75250	0.75825
Top5	Avg	0.75225	0.74725	0.74894	0.74980	0.75344	0.75260	0.75219	0.75532
Top 10	Avg	0.75144	0.74682	0.74658	0.74923	0.75274	0.75179	0.75194	0.75435
Top 50	Avg	0.74852	0.7456	0.74658	0.74767	0.75114	0.75031	0.75091	0.75206
Top 100	Avg	0.74692	0.7449	0.74584	0.74704	0.75044	0.74956	0.75038	0.75122
All	Avg	0.66456	0.6839	0.72096	0.73505	0.73657	0.72052	0.71997	0.63960

**Table 5:** Comparison of group prediction performance in average mean_test_AUCs for the DFNN-10-year models.

Group	Results	Stage1	Stage2	Stage3-c1	Stage3-c2	Stage3-c3	Stage3-c4	Stage3-c5	Stage3-c6
Top1	best	0.77789	0.78280	0.78802	0.79023	0.79152	0.78885	0.78059	0.78564
Top5	Avg	0.77593	0.78141	0.78696	0.78897	0.79030	0.78762	0.77936	0.78272
Top10	Avg	0.77511	0.78090	0.78626	0.78820	0.78980	0.78703	0.77889	0.78189
Top50	Avg	0.77348	0.77942	0.78475	0.78644	0.78865	0.78553	0.77731	0.77992
Top100	Avg	0.77257	0.77866	0.78402	0.78551	0.78797	0.78483	0.77652	0.77900
All	Avg	0.71344	0.73360	0.76124	0.75901	0.76702	0.76194	0.74336	0.68075

**Table 6:** Comparison of group prediction performance in average mean_test_AUCs for the DFNN-15-year models.

Group	Results	Stage1	Stage2	Stage3-c1	Stage3-c2	Stage3-c3	Stage3-c4	Stage3-c5	Stage3-c6
Top1	best	0.86502	0.86548	0.88023	0.87463	0.87359	0.87551	0.87255	0.86657
Top5	Avg	0.86232	0.86403	0.87632	0.87397	0.87337	0.87476	0.87095	0.86578
Top 10	Avg	0.86105	0.86294	0.87426	0.87353	0.87280	0.87427	0.87028	0.86510
Top 50	Avg	0.85806	0.86069	0.87073	0.87149	0.87082	0.87281	0.86872	0.86271
Top 100	Avg	0.85678	0.85966	0.86950	0.87055	0.86954	0.87199	0.86789	0.86170
All	Avg	0.76512	0.78853	0.83167	0.84535	0.83399	0.83782	0.83731	0.75042

**Table 7:** Mid-point group results of grid searches.

		Stage1	Stage2	Stage3-c1	Stage3-c2	Stage3-c3	Stage3-c4	Stage3-c5	Stage3-c6
5 Y	Best-mean	0.75301	0.74812	0.75024	0.75066	0.75418	0.75386	0.75250	0.75825
	Mid-point	0.62650	0.62406	0.62512	0.62533	0.62709	0.62693	0.62625	0.62913
	CHS	39,112	510,494	203,991	101,250	202,500	404,726	408,625	43,035
	TNS	52,042	582,471	202,500	101,250	202,500	405,000	409,050	70,568
	CHS/TNS	0.75155	0.87643	0.99946	1.0	1.0	0.99932	0.99896	0.60984
	Avg-mean	0.71116	0.70278	0.72102	0.73505	0.73657	0.72059	0.72010	0.70415
10 Y	Best_mean	0.77789	0.78280	0.78802	0.79023	0.79152	0.78885	0.78059	0.78564
	Mid_point	0.63895	0.64140	0.64401	0.64512	0.64576	0.64442	0.64029	0.64282
	CHS	48,517	635,770	202,500	60,750	202,495	405,000	404,994	83,896
	TNS	52,416	638,244	202,500	60,750	202,500	405,000	405,000	105,500
	CHS/TNS	0.92561	0.99612	1.0	1.0	0.99998	1.0	0.99999	0.79522
	Avg_mean	0.72116	0.73400	0.76124	0.75901	0.76702	0.76194	0.74337	0.72642
15 Y	Best_mean	0.86502	0.86548	0.88023	0.87463	0.87359	0.87551	0.87255	0.86657
	Mid_point	0.68251	0.68274	0.69011	0.68731	0.68679	0.68776	0.68628	0.68328
	CHS	41,065	952,677	337,492	162,000	270,000	2,058,750	405,000	73,007
	TNS	52,090	1,048,575	337,500	162,000	270,000	2,058,750	405,000	93,000
	CHS/TNS	0.78835	0.90854	0.99998	1.0	1.0	1.0	1.0	0.78502
	Avg_mean	0.800615	0.80289	0.83167	0.84535	0.83399	0.83782	0.83731	0.81619

Best-mean: the highest mean_test_AUC of all corresponding models; mid-point: the average of 0.5 and the best-mean AUC; CHS: the count of hyperparameter settings at which the mean_test_AUCs are at least as high as the corresponding mid-point AUC; TNS: the total number of hyperparameter settings tested during the corresponding cycle of grid searches; CHS/TNS: the ratio of CHS over TNS; avg-mean: the average mean_test_AUC of all models no worse than the mid-point AUC; Y: year.

**Table 8:** Running time and count of hyperparameter settings concerning grid searches.

Stage	5-Year			10-Year			15-Year		
	RTPS	TNS	TRT	RTPS	TNS	TRT	RTPS	TNS	TRT
Stage1	42.42	52,042	613.23	26.57	52,416	386.87	20.06	52,090	290.25
Stage2	5.65	582,471	914.72	3.36	638,244	594.86	2.49	1,048,575	725.06
Stage3-c1	10.65	202,500	599.01	3.55	202,500	199.42	2.43	337,500	227.39
Stage3-c2	11.97	101,250	336.54	5.49	60,750	92.66	1.63	162,000	73.17
Stage3-c3	11.11	202,500	624.88	6.50	202,500	365.77	1.25	270,000	93.60
Stage3-c4	25.46	405,000	2863.73	7.66	405,000	861.75	1.89	2,058,750	1080.01
Stage3-c5	66.80	409,050	7590.07	29.44	405,000	3311.76	12.22	398,252	1352.36
Stage3-c6	116.40	70,568	2281.77	51.05	105,500	1495.91	27.36	93,000	706.71

RTPS: running time per hyperparameter setting in seconds; TNS: total number of hyperparameter settings; TRT: total running time in hours.

## Data Availability

The data used in this study are available at datadryad.org (DOI 10.5061/dryad.64964m0).

## Supplementary Materials

The following supporting information can be downloaded at: https://www.mdpi.com/article/10.3390/cancers17071092/s1, Table S1: Predictors in the LSM_RF-5Year Dataset; Table S2: Predictors in the LSM_RF-10Year Dataset; Table S3: Predictors in the LSM_RF-15 Year Dataset; Table S4: Range of hyperparameter values used in the three-stage grid searches for predicting 5 year breast cancer metastasis; Table S5: Range of hyperparameter values used in the three-stage grid searches for predicting 10 year breast cancer metastasis; Table S6: Range of hyperparameter values used in the three-stage grid searches for predicting 15 year breast cancer metastasis.
