# Supplementary material for: Deep Learning: A Heuristic Three-Stage Mechanism for Grid Searches to Optimize the Future Risk Prediction of Breast Cancer Metastasis Using EHR-Based Clinical Data
Source: Cancers (Basel). 2025 Mar 25;17(7):1092. doi: 10.3390/cancers17071092 (PMC11987998; doi:10.3390/cancers17071092)
Supplement: Supplementary file 1 [file cancers-17-01092-s001.zip › cancers-3471283-supplementary.pdf]

Table S1: Predictors in the LSM_RF-5Year Dataset

	Predictors	Description	Values
1	race	race of patient	white, black, Asian, American Indian or Alaskan native, native Hawaiian or other Pacific islander
2	smoking	smoking history of patient	ex smoker, non smoker, cigarettes, chewing tobacco, cigar
3	family history	family history of cancer	cancer, no cancer, breast cancer, other cancer, cancer but nos
4	age_at_diagnosis	age at diagnosis of the disease	0-49, 50-69, >69
5	TNEG	triple negative status in terms of patient being ER, PR, and HER2 negative	yes, no
6	ER	estrogen receptor expression	neg, pos, low pos
7	ER_percent	percent of cell stain pos for ER receptors	0-20, 20-90, 90-100
8	PR	progesterone receptor expression	neg, pos, low pos
9	PR_percent	percent of cell stain pos for PR receptors	0-20, 20-90, 90-100
10	P53	P53	whether P53 is mutated
11	HER2	HER2 expression	neg, pos
12	t_tnm_stage	prime tumor stage in TNM system	0, 1, 2, 3, 4, IS, 1mic, X
13	n_tnm_stage	# of nearby cancerous lymph nodes	0, 1, 2, 3, 4, X
14	stage	composite of size and # positive nodes	0, 1, 2, 3
15	lymph_nodes_positive	number of positive lymph nodes	0, 1-8, >8
16	histology	tumor histology	lobular, duct
17	size	size of tumor in mm	0-32, 32-70, >70
18	invasive_tumor_location	where invasive tumor is located	mixed duct and lobular, duct, lobular, none
19	DCIS_level	type of ductal carcinoma in situ	solid, apocrine, cribriform, dcis, comedo, papillary, micropapillary
20	surgical_margins	whether residual tumor	res. tumor, no res. tumor, no primary site surgery

Table S2. Predictors in the LSM_RF-10Year Dataset

	Predictors	Description	Values
1	ethnicity	ethnicity of patient	not Hispanic, Hispanic
2	smoking	smoking history of patient	ex smoker, non smoker, cigarettes, chewing tobacco, cigar
3	alcohol usage	alcohol usage of patient	moderate, no use, use but nos (non otherwise specified), former user, heavy user
4	family history	family history of cancer	cancer, no cancer, breast cancer, other cancer, cancer but nos

5	age_at_diagnosis	age at diagnosis of the disease	0-49, 50-69, >69
6	TNEG	triple negative status in terms of patient being ER, PR, and HER2 negative	yes, no
7	ER	estrogen receptor expression	neg, pos, low pos
8	ER_percent	percent of cell stain pos for ER receptors	0-20, 20-90, 90-100
9	PR	progesterone receptor expression	neg, pos, low pos
10	PR_percent	percent of cell stain pos for PR receptors	0-20, 20-90, 90-100
11	HER2	HER2 expression	neg, pos
12	n_tnm_stage	# of nearby cancerous lymph nodes	0, 1, 2, 3, 4, X
13	stage	composite of size and # positive nodes	0, 1, 2, 3
14	lymph_nodes_positive	number of positive lymph nodes	0, 1-8, >8
15	histology	tumor histology	lobular, duct
16	grade	grade of disease	1, 2, 3
17	DCIS_level	type of ductal carcinoma in situ	solid, apocrine, cribriform, dcis, comedo, papillary, micropapillary
18	surgical_margins	whether residual tumor	res. tumor, no res. tumor,<br>no primary site surgery

Table S3: Predictors in the LSM_RF-15 Year Dataset

	Predictors	Description	Values
1	race	race of patient	white, black, Asian, American Indian or Alaskan native, native Hawaiian or other Pacific islander
2	alcohol usage	alcohol usage of patient	moderate, no use, use but nos (non otherwise specified), former user, heavy user
3	age_at_diagnosis	age at diagnosis of the disease	0-49, 50-69, >69
4	menopausal_status	inferred menopausal status	pre, post
5	ER	estrogen receptor expression	neg, pos, low pos
6	ER_percent	percent of cell stain pos for ER receptors	0-20, 20-90, 90-100
7	t_tnm_stage	prime tumor stage in TNM system	0, 1, 2, 3, 4, IS, 1mic, X
8	n_tnm_stage	# of nearby cancerous lymph nodes	0, 1, 2, 3, 4, X
9	stage	composite of size and # positive nodes	0, 1, 2, 3
10	lymph_node_status	patient had any positive lymph nodes	neg, pos
11	size	size of tumor in mm	0-32, 32-70, >70
12	grade	grade of disease	1, 2, 3
13	histology2	tumor histology subtypes	IDC, DCIS, ILC, NC

14	invasive_tumor_location	where invasive tumor is located	mixed duct and lobular, duct, lobular, none
15	re_excision	removal of an additional margin of tissue	yes, no
16	surgical_margins	whether residual tumor	res. tumor, no res. tumor,<br>no primary site surgery
17	histology	tumor histology	lobular, duct

Table S4. Range of hyperparameter values used in the three-stage grid searches for predicting 5 year breast cancer metastasis

Hyperparameter Name	Hyperparameter Values							
	Stage 1	Stage 2	Stage 3-c1	Stage 3-c2	Stage 3-c3	Stage 3-c4	Stage 3-c5	Stage 3-c6
# of Hidden Layers	1,2,3,4	1,2,3,4	2,3,4	4	2,3,4	2,3,4	2,3,4	1,2,3,4
# of Hidden Nodes Each Layer	1 to 1005	1 to 350	100 to 350	100 to 200	100 to 350	200 to 350	350 to 550	550 to 800
Activation Function	'relu'	'relu'	'relu'	'relu'	'relu'	'relu'	'relu'	'relu'
Kernel initializer	'he_normal', 'glorot_normal'	'Glorot_normal'	'Glorot_normal'	'Glorot_normal'	'Glorot_normal'	'Glorot_normal'	'Glorot_normal'	'Constant', 'Glorot_normal', 'Glorot_uniform', 'He_normal', 'He_uniform'
Optimizer	'SGD', 'Adagrad'	'Adagrad'	'Adagrad'	'Adagrad'	'Adagrad'	'Adagrad'	'Adagrad'	'SGD','Adagrad','adam'
Learning rate	0.001 to 0.3	0.01 to 0.1	0.01 to 0.09	0.05 to 0.09	0.07 to 0.09	0.01 to 0.09	0.01 to 0.09	0.01 to 0.09
Momentum	0 to 0.9	0 to 0.4	0.1 to 0.4	0.1 to 0.2	0.12 to 0.18	0.1 to 0.4	0.1 to 0.4	0.1 to 0.9
Iteration-based Decay	0 to 0.1	0 to 0.001	0 to 0.0005	0 to 0.0006	0.0004 to 0.0006	0 to 0.0005	0 to 0.1	0 to 0.001
Dropout rate	0 to 0.5	0 to 0.2	0 to 0.1	0 to 0.05	0.03 to 0.05	0 to 0.1	0 to 0.1	0 to 0.1
Epochs	5 to 2000	25 to 175	100 to 180	140 to 180	160 to 180	100 to 180	100 to 180	100 to 180
Batch_size	1 to 4189	100 to 1000	100 to 500	300 to 540	460 to 540	200 to 500	100 to 500	100 to 500

L1	0 to 0.03	0 to 0.01	0 to 0.01	0.004 to 0.006	0.004 to 0.005	0 to 0.01	0 to 0.01	0 to 0.03
L2	0 to 0.2	0 to 0.05	0 to 0.01	0 to 0.01	0 to 0.005	0	0	0 to 0.03

Table S5. Range of hyperparameter values used in the three-stage grid searches for predicting 10 year breast cancer metastasis

Hyperparameter Name	Hyperparameter Values							
	Stage 1	Stage 2	Stage 3-c1	Stage 3-c2	Stage 3-c3	Stage 3-c4	Stage 3-c5	Stage 3-c6
# of Hidden Layers	1,2,3,4	1,2,3,4	2,3,4	4	2,3,4	2,3,4	2,3,4	1,2,3,4
# of Hidden Nodes Each Layer	5 to 1005	50 to 200	50 to 200	50 to 150	50 to 200	50 to 200	350 to 550	550 to 800
Activation Function	‘relu’	‘relu’	‘relu’	‘relu’	‘relu’	‘relu’	‘relu’	‘relu’
Kernel initializer	‘he_normal’, ‘glorot_normal’	‘Glorot_normal’	‘Glorot_normal’	‘Glorot_normal’	‘Glorot_normal’	‘Glorot_normal’	‘Glorot_normal’	‘Constant’, ‘Glorot_normal’, ‘Glorot_uniform’, ‘He_normal’, ‘He_uniform’
Optimizer	‘SGD’, ‘Adagrad’	‘Adagrad’	‘Adagrad’	‘Adagrad’	‘Adagrad’	‘Adagrad’	‘Adagrad’	‘SGD’,’Adagrad’,’adam’
Learning rate	0.001 to 0.299	0.01 to 0.2	0.01 to 0.1	0.03 to 0.05	0.025 to 0.29	0.01 to 0.1	0.01 to 0.09	0.01 to 0.09
Momentum	0 to 0.9	0.4	0.1 to 0.4	0.1 to 0.2	0.1 to 0.15	0.1 to 0.4	0.1 to 0.4	0.1 to 0.9
Iteration-based Decay	0 to 0.01	0 to 0.001	0 to 0.001	0.0006 to 0.001	0.0009 to 0.0011	0 to 0.001	0 to 0.0005	0 to 0.001
Dropout rate	0 to 0.5	0 to 0.2	0.1 to 0.2	0.1 to 0.25	0.21 to 0.24	0.1 to 0.2	0 to 0.1	0 to 0.1
Epochs	5 to 1960	10 to 160	110 to 150	150 to 190	165 to 185	110 to 150	100 to 180	100 to 180
Batch_size	1 to 1827	40 to 250	40 to 250	40 to 120	30 to 90	40 to 250	100 to 500	100 to 500
L1	0 to 0.199	0 to 0.01	0.002 to 0.005	0 to 0.003	0.002 to 0.004	0.002 to 0.005	0 to 0.01	0 to 0.03
L2	0 to 0.199	0 to 0.02	0 to 0.005	0 to 0.008	0 to 0.003	0	0	0 to 0.03

Table S6. Range of hyperparameter values used in the three-stage grid searches for predicting 15 year breast cancer metastasis

Hyperparameter Name	Hyperparameter Values							
	Stage 1	Stage 2	Stage 3-c1	Stage 3-c2	Stage 3-c3	Stage 3-c4	Stage 3-c5	Stage 3-c6
# of Hidden Layers	1,2,3,4	1,2,3,4	3,4	3,4	3,4	2,3,4	2,3,4	1,2,3,4
# of Hidden Nodes Each Layer	5 to 1005	50 to 150	50 to 110	50 to 110	50 to 110	50 to 140	350 to 550	550 to 800
Activation Function	'relu'	'relu'	'relu'	'relu'	'relu'	'relu'	'relu'	'relu'
Kernel initializer	'he_normal', 'glorot_normal'	'Glorot_normal'	'Glorot_normal'	'Glorot_normal'	'Glorot_normal'	'Glorot_normal'	'Glorot_normal'	'Constant', 'Glorot_normal', 'Glorot_uniform', 'He_normal', 'He_uniform'
Optimizer	'SGD', 'Adagrad'	'Adagrad'	'Adagrad'	'Adagrad'	'Adagrad'	'Adagrad'	'Adagrad'	'SGD','Adagrad','adam'
Learning rate	0.001 to 0.299	0.01 to 0.1	0.01 to 0.1	0.03 to 0.07	0.03 to 0.05	0.01 to 0.1	0.01 to 0.09	0.01 to 0.09
Momentum	0 to 0.9	0.4	0.1 to 0.4	0.3 to 0.5	0.35 to 0.45	0.1 to 0.4	0.1 to 0.4	0.1 to 0.9
Iteration-based Decay	0 to 0.01	0 to 0.001	0 to 0.001	0 to 0.0005	0 to 0.0002	0 to 0.001	0 to 0.0005	0 to 0.001
Dropout rate	0 to 0.5	0 to 0.2	0 to 0.2	0 to 0.1	0 to 0.05	0 to 0.2	0 to 0.1	0 to 0.1
Epochs	5 to 1960	5 to 95	50 to 95	50 to 90	70 to 110	50 to 95	100 to 180	100 to 180
Batch_size	1 to 751	60 to 200	60 to 200	300 to 540	50 to 110	60 to 200	100 to 500	100 to 500
L1	0 to 0.199	0 to 0.025	0 to 0.002	0 to 0.003	0.00035 to 0.00045	0 to 0.002	0 to 0.01	0 to 0.03
L2	0 to 0.199	0 to 0.025	0 to 0.025	0 to 0.005	0.0015 to 0.003	0 to 0.025	0	0 to 0.03
